# Efficacy to effectiveness transition of an Educational Program to Increase Colorectal Cancer Screening (EPICS): study protocol of a cluster randomized controlled trial

**DOI:** 10.1186/1748-5908-8-86

**Published:** 2013-08-07

**Authors:** Selina A Smith, Daniel S Blumenthal

**Affiliations:** 1 Department of Community Health and Preventive Medicine, Morehouse School of Medicine, Atlanta, GA, USA; 2 Cancer Research Program, Morehouse School of Medicine, Atlanta, GA, USA

**Keywords:** Dissemination, Implementation, Colorectal cancer screening, Health disparities, Community-based participatory research

## Abstract

**Background:**

African Americans have the highest incidence and mortality and are less likely than whites to have been screened for colorectal cancer (CRC). Many interventions have been shown to increase CRC screening in research settings, but few have been evaluated specifically for use in African-American communities in real world settings. This study aims to identify the most efficacious approach to disseminate an evidence-based intervention in promoting colorectal screening in African Americans and to identify the factors associated with its efficacy.

**Methods/design:**

In this study, investigators will recruit 20 community coalitions and 7,200 African-Americans age 50 to 74 to test passive and active approaches to disseminating the Educational Program to Increase Colorectal Cancer Screening (EPICS); to measure the extent to which EPICS is accepted and the fidelity of implementation in various settings and to estimate the potential translatability and public health impact of EPICS. This four-arm cluster randomized trial compares the following implementation strategies: passive arms, (web access to facilitator training materials and toolkits without technical assistance (TA) and (web access, but with technical assistance (TA); active arms, (in-person access to facilitator training materials and toolkits without TA and (in-person access with TA). Primary outcome measures are the reach (the proportion of representative community coalitions and individuals participating) and efficacy (post-intervention changes in CRC screening rates). Secondary outcomes include adoption (percentage of community coalitions implementing the EPICS sessions) and implementation (quality and consistency of the intervention delivery). The extent to which community coalitions continue to implement EPICS post-implementation (maintenance) will also be measured. Cost-effectiveness analysis will be conducted.

**Discussion:**

Implementing EPICS in partnership with community coalitions, we hypothesized, will result in more rapid adoption than traditional top-down approaches, and resulting changes in community CRC screening practices are more likely to be sustainable over time. With its national reach, this study has the potential to enhance our understanding of barriers and enablers to the uptake of educational programs aimed at eliminating cancer disparities.

**Trial registration:**

http://www.ClinicalTrials.gov NCT01805622

## Background

Colorectal cancer (CRC) is the third most commonly diagnosed cancer and the second leading cause of cancer death in the United States (US) [1]. The American Cancer Society (ACS) estimates that over 142,820 people will be diagnosed with CRC and over 50,830 people will die from this disease in 2013 [1]. The most frequently recommended screening tests for CRC include annual fecal occult blood testing (FOBT) or colonoscopy every ten years. Alternative screening tests are sometimes used: sigmoidoscopy every five years (combined with annual FOBT), or double-contrast barium enema every five years. Fecal DNA and computed colonoscopy are available, but still considered investigational by many third-party payers. Endoscopy is not only a mode of early detection (secondary prevention); it also serves as primary prevention when a precancerous polyp is removed. CRC screening is substantially underutilized in the US, with only about 55% of those for whom screening is recommended (persons 50 years and older) having been screened with the recommended frequency [2].

Factors associated with the use of CRC screening may be characterized as patient-related, physician-related, or system-related. Patient-related factors include having insurance coverage, a source of medical care, and higher education and income. A number of knowledge, belief, and attitudinal factors also play an important role [3]. A screening recommendation from a physician is the only physician-related factor. System-related factors include electronic medical records, ancillary personnel who can provide follow-up and patient navigators [2]. African Americans have by far the highest CRC incidence and mortality rates of any racial/ethnic group. African American mortality is 50% higher than the second-highest group (white), and more than double that of the group with the lowest mortality (Asian/Pacific Islander) [4]. Likely related to the mortality disparity is the disparity in CRC screening. Similar percentages of African Americans (23.8%) and whites (21.3%) age 50 years and older have had an FOBT within the last two years, but whites (64.0%) are substantially more likely to have had endoscopy (ever) than African Americans (58.6%) (2008 data) [5].

Given the high mortality rate, the substantial racial disparity and the reluctance of Americans (especially African Americans) to be screened, the dissemination of an evidence-based, low-cost intervention that would increase screening is highly significant.

In 2010, we published the results of the CRC Screening Intervention Trial (CCSIT) [6] developed using community-based participatory research principles [7]. We partnered with and received input from a community coalition of advocates and agency staff. That coalition and the Community Coalition Board of the Morehouse Prevention Research Center (similar to a community advisory board) also participated in carrying out the project, and the latter group assisted in interpreting and disseminating results. This was a randomized controlled community intervention trial in which three hundred and sixty-nine African American men and women (aged ≥50) in the Atlanta, GA metropolitan area were randomized to participate in one of three interventions that had been chosen to address evidence gaps in the Guide to Community Preventive Services [8]: one-on-one education, group education, and reducing out-of-pocket costs. We compared the three experimental cohorts to a control cohort. Following the interventions, there were significant increases in knowledge about CRC in both educational cohorts but in neither the reduced out-of-pocket cost nor the control cohort. By the six-month follow-up, 17.7% (11/6 of control group members reported having undergone screening, as compared to 33.9% (22/6 of the small group education (p = 0.039), 25.4% (17/6 of the one-on-one education (p = ns), and 22.2% (14/6 of the reduced out-of-pocket cost groups (p > 0.05).

The intervention included four one-hour sessions, each consisting of a small group led by a health educator or community health worker (facilitators). The presentations and discussion covered CRC mortality data, signs and symptoms, modifiable risk factors (*e.g.*, dietary intake), screening tests, and treatment approaches.

Subsequent to CCSIT, we entered into a partnership with the local health department (Fulton County Department of Health and Wellness) and local Office on Aging to put EPICS into practice in the county’s 15 senior citizen centers. At the urging of the health department’s health educators, the number of sessions in the intervention was shortened to three. In the practice demonstration, 331 mostly African-American men and women 50 years and older who had never been screened for CRC, or who were not up-to-date, received the intervention. At three-month follow-up, 37.3% had been screened; another 33.8% stated that they had appointments for screening or intended to get an appointment [9]. It is unusual for an intervention to perform as well in practice (effectiveness) as in the research setting (efficacy). However, EPICS performed at least as well in practice as it did in the community intervention trial, even discounting participants who had not yet been screened, but stated that they intended to do so or had an appointment [9].

Based on initial success in a ‘real world’ setting, we then initiated a state-wide EPICS dissemination and implementation project. To reach most of the state, we partnered with five of the six Regional Cancer Coalitions of Georgia (RCCG) and their parent body, the Georgia Cancer Coalition (GCC). We trained facilitators in each of the regional coalitions and in 12 hospital-based American College of Surgeon-accredited cancer programs. We also gained firsthand knowledge of dissemination and implementation processes from the state practice demonstration that will be used in the proposed project, including strategies for: engaging decision makers and stakeholders; developing, packaging, and enhancing the intervention toolkit; designing and improving facilitator training and certification; developing an implementation protocol; establishing quality assurance measures; and enhancing technical assistance for successful efficacy-to-effectiveness translation [10].

### Goals and aims

The overall goals of this research are to identify the most efficacious approach to disseminating EPICS and to identify the factors associated with its effectiveness.

Key aims are to:

1. Test passive and active approaches to disseminating EPICS.

2. Measure the extent to which EPICS is accepted by community coalitions and the fidelity of implementation in various settings.

3. Estimate the potential for translation and the public health impact of EPICS.

## Methods/design

### Ethical approval and informed consent

The Institutional Review Board (IRB) of the Morehouse School of Medicine has approved our research plan. Informed consent will be obtained for all study participants: community coalition leaders, EPICS facilitators, and individual participants. The study is registered with ClinicalTrials.gov as NCT01805622.

### Community coalitions

The community coalitions that will serve as clusters in the EPICS cRCT are part of the National Black Leadership Initiative on Cancer (NBLIC), a network that was founded in 1987 and have primarily engaged in community education efforts; this history has been described elsewhere [11]. The coalitions include individuals from academia, business, health care, non-profit organizations, and other community stakeholders. Twenty of the 33 NBLIC community coalitions will be randomized as clusters in the EPICS cRCT. In developing this project, we executed Memoranda of Understanding (MOU) between the coalitions and Morehouse School of Medicine; recruited research sites and surveyed the community coalitions’ organizational characteristics.

### Trial design

**Figure 1:**
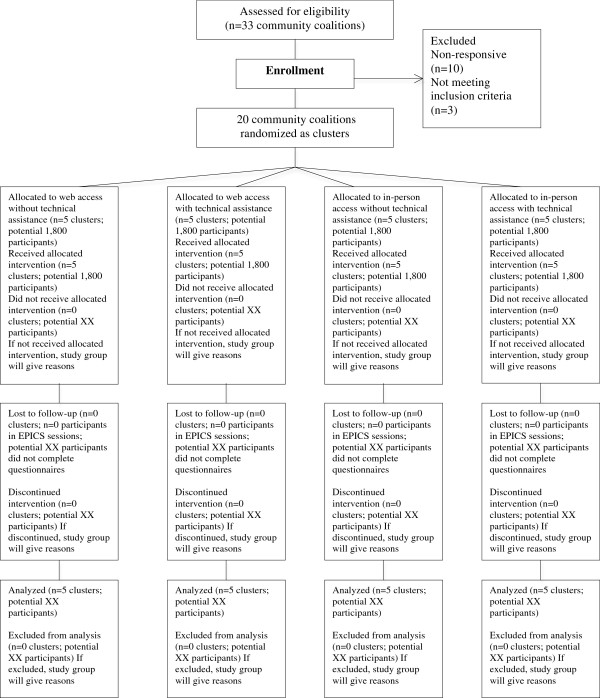
CONSORT flow diagram for EPICS cRCT.

This study is a four-arm, cluster randomized controlled trial (cRCT). Figure 1 depicts information from four stages of the EPICS cRCT (enrollment, intervention allocation, follow-up, and analysis) based on the recommended flow of parallel group randomized trials [12].

Randomization at the organizational (community coalition) level has been chosen because this will minimize contamination that may occur if individual participants are randomized. In utilizing cluster randomization, we will focus on community coalitions as the cluster for all study arms, with individuals within their target settings as the secondary unit. We will ensure balance in all four study arms by ordering them into groups based on the total number participating. A computer program generating random numbers between 1 and *n* will be used to assign community coalitions to one of four conditions: web access (WA) to facilitator training materials and toolkits without technical assistance (TA); the same as 1, but with technical assistance (TA); in-person access (IP) to facilitator training materials and toolkits without TA; and in-person access to facilitator training materials and toolkits with TA. Our multilevel approach (*e.g.*, cluster, setting, and individual) promotes widespread dissemination [13]. Community coalitions will be allocated to one of the four arms and informed consent will be obtained from community coalition members and from individual participants.

### Toolkit

The EPICS toolkit is a package that includes facilitator training materials (manual, workshop agenda, and certificates); implementation protocol; EPICS curriculum; individual/participant educational materials; and individual/participant incentives. The toolkit was developed and pilot tested during the local and state practice demonstrations. Community coalitions randomized to passive dissemination arms will access all toolkit components except participant incentives via the web. Those randomized to active dissemination will receive all toolkit components via in-person delivery. In the case of individual/participant incentives, delivery will be via US Postal Service for community coalitions in all dissemination arms.

1. Facilitator training: Consistent with principles of Adult Learning Theory, the EPICS facilitator training workshop is skill-based, highly participatory, and provides learners with immediate opportunities to apply new skills and information [14]. A user-friendly Facilitator Training Manual has been developed and tested. The one-and-one-half-day facilitator training workshop introduces basic vocabulary, concepts, and methods of community-based cancer control and instructional strategies for African Americans of varying health literacy. Each community coalition will identify a minimum of five members, in addition to a leader, to complete facilitator training. Community coalitions randomized to passive dissemination arms will download training materials from the National Cancer Institute’s Research Tested Intervention Programs (RTIPs) website (http://rtips.cancer.gov/rtips/index.do) and conduct the training themselves, while those in active dissemination arms will be trained by EPICS developers. The facilitator training workshop consists of three modules that address: principles knowledge (*e.g.*, conceptual framework, intervention overview, and local/statewide demonstration projects); procedural knowledge (*e.g.*, how to implement the three EPICS sessions); and practical knowledge (*e.g.*, how to market the programs and complete the EPICS assessments and measures).

2. Implementation Protocol: We also developed and tested a protocol similar to that used in the original CCSIT study and converted it into non-academic language. This 12-step protocol includes procedures to: recruit community partners; schedule sessions; market the program; recruit participants; prepare for the sessions; sort the toolkit; assign facilitators; deliver the sessions; identify ‘completers’ (participants attending all three intervention sessions); monitor screening; submit reports; and get technical assistance.

3. EPICS Curriculum: Individual participants in all study arms will complete three small group educational sessions. These sessions, each approximately one-hour in length, will be conducted the same day/time of the week for three consecutive weeks. This schedule will be formulated by NBLIC community coalitions in conversation with intervention settings at a time likely to solicit the greatest participation.

4. Individual/participant CRC educational materials include three session-specific brochures, CDC consumer education materials, including: Get Smart in Your Family Dinner; Get Smart as You Shop; and CRC Facts on Screening.

5. Individual/participant incentives such as a cookbook, water bottles, shopping bag, and similar items are distributed to individuals completing all three EPICS sessions.

### Churches, clinical partners, and community sites

Three settings with congregants, patients or clients, aged 50 to 74 years, will participate in the study: churches, including Protestant and Catholic churches and other faith-based institutions; clinical cites, including health departments, community health centers, hospitals and similar healthcare providers; and community cites*,* including senior citizen centers, assisted living facilities, and community centers. NBLIC community coalitions, based on their relationship and history with these community institutions, will work closely with the leaders of these organizations to assist in individual participant recruitment.

### Individual participants

English-speaking, African Americans, 50 to 74 years of age, who are not current on CRC screening, are eligible for study participation. Individuals with a personal history of CRC or inflammatory bowel disease, blindness or severe hearing impairment; dementia; or other condition with life expectancy less than two years, are ineligible for participation.

### Sample size calculation

We intend to enroll 7,200 individuals in this study, with 1,800 enrolled in each study arm. This sample size is based on 20 NBLIC community coalitions randomized to four study arms (WA, WA + TA, IP, IP + TA). There will also be three settings (church, clinic, and community site) per coalition. Each community coalition will have 30 groups of individuals completing three consecutive EPICS sessions, recruiting 15 individuals per setting with an expected 25% dropout rate; thus, we anticipate that 12 of these individuals will complete all three sessions. For the EPICS cRCT, there will be (30 groups of three sessions) × (12 individuals completing all three sessions) × (20 NBLIC community coalitions) = 7,200 individuals. Based on data from a previously published study, we calculated intracluster correlation coefficient (ICC) to equal 0.0911 (9.1%) [6]. The effective sample size (ESS) was 5,645, which still has high power (>99%) with design effect of 1.2754.

### Hypotheses

We propose three hypotheses to measure the impact of our effort:

Hypothesis one (H1): When compared to passive dissemination, active dissemination will result in greater participant enrollment.

Hypothesis two (H2): The intervention will be offered with equal fidelity in churches, clinics, and community sites.

Hypothesis three (H3): Knowledge of CRC screening and perceived risk of CRC will be positively correlated.

### Power analysis

Power calculations were performed to further justify our proposed sample size. All of these assume a sample size of 1,800 per study arm, a significance level of 5% (*i.e.* alpha = 0.05), and a two-sided two-group chi-square test of proportions. Calculations were performed using nQuery Advisor, version 7. These are performed for hypothesis one (H1) and hypothesis three (H3); hypothesis two (H2) is qualitative, so power calculations do not apply. For H1, we anticipate that there will be 25%, 33%, and 50% greater participant enrollment with the Web + TA arm, IP arm, and IP + TA arm, respectively, when compared to the passive web-based-only arm (based on our prior experience with community coalition based studies). Comparisons of any of these proportions to any proportion that is obtained for the passive web-based arm (for example, 5%, 10%, or 20%) will result in greater than 99% power to detect differences in the proportions as being statistically significant. For H3, we can obtain estimates of cancer knowledge change over time (pre-intervention to post-intervention percentages with the correct response) for a small group education cohort and a control cohort from Blumenthal, Smith *et al.*[6]. These estimates include changes of 34.8 (71-36) and 15.5 (55.2-39) for a CRC perceived risk question, and 22.9 (92.9–70) and 13.1 (88.5–75.for a CRC screening question. Assuming that we observe similar changes in our study participants for active and passive arms, we will have greater than 99% power to detect differences in the proportions as being statistically significant.

### Theoretical framework

**Figure 2:**
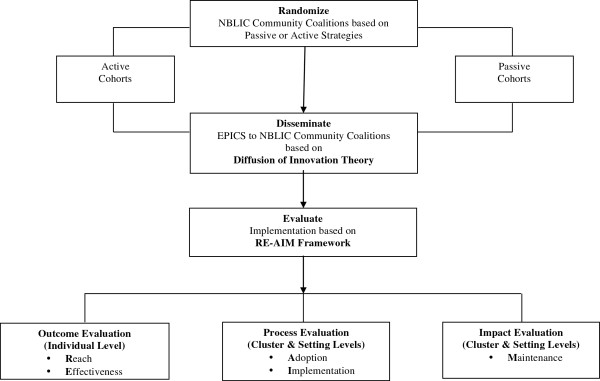
Study overview.

We will use Rogers’ Diffusion of Innovation Theory and Glasgow’s RE-AIM (Reach, Effectiveness, Adoption, Implementation, Maintenance) evaluation framework to guide the EPICS cRCT (Figure 2).

Based on Rogers’ theory, innovations that are simple, such as EPICS, are often adopted more rapidly and lead to a higher degree of sustainability. Diffusion of Innovations Theory addresses the process of adoption and uptake of interventions by a targeted audience. Three key components of this theory make it well-suited to facilitate adoption of EPICS at the cluster and setting levels. Research suggests that the most effective communication strategy is face-to-face exchange [15]. We hypothesized that social support theory, which uses informal communication channels, helped explain the success of EPICS.

We will continue to use informal communication channels (as recommended by Rogers) to reach change agents and early adopters. Change agents are organizations connected to early adopters*,* organizations that will attempt EPICS before other organizations [15]. Rogers’ theory will provide valuable insights into why EPICS is adopted by some NBLIC community coalitions and not by others. In the EPICS cRCT, this theory will:

1. Explain variance in adoption rates: All clusters will complete Barriers and Enablers to Uptake Assessment (which include setting level-specific questions) to measure key attributes.

2. Explore the impact of facilitators on EPICS dissemination: We will compare key attributes and facilitator knowledge, attitudes and confidence (Pre and Post-Facilitator Training Knowledge, Attitudes and Confidence survey).

3. Evaluate fidelity of implementation to determine adherence to EPICS core elements.

4. Examine the relationship between adoption and outcomes: (*e.g.*, cost-effectiveness analysis, CRC screening rates and number of individuals completing all three EPICS sessions).

5. Establish the role of TA*:* Comparing passive and active arms (providing reinforcement and positive feedback in Rogers’ confirmation stage).

RE-AIM is a framework developed by Glasgow *et al.* designed to evaluate interventions that change health behavior [16]. It has been applied to dissemination studies of various behavioral modification interventions, including a smokeless tobacco intervention [17], diabetes self-management programs [18], smoking cessation [19], physical activity [20], and worksite health behavior interventions [21], as well as interventions in churches [22] and schools [23]. The RE-AIM model is intended to refocus priorities on public health issues and provide balanced emphasis on internal and external validity. Glasgow also concluded that simple and flexible interventions that can be implemented by non-researchers have the greatest potential for long-term maintenance.

RE-AIM is a good tool for evaluating the dissemination of interventions like EPICS, which have the potential to reach a large audience, can be broadly adopted, and can be implemented by different types of staff without a heavy investment of resources [16].

### Primary outcome measures

The primary outcome for the EPICS cRCT focuses on the Reach and Effectiveness components of the RE-AIM (Reach, Effectiveness, Approach, Implementation, Maintenance) model. Reach refers to the proportion of representative eligible organizations, settings, and individuals participating in the intervention. To determine EPICS Reach, we will examine two dimensions of this concept, estimated based on the following calculation [24]:

Number of participants in

$$\text{Estimate}\;\text{of}\;\text{REACH}=\frac{\text{the}\;\text{target}\;\text{population}\;\text{who}\;\text{receive}\;\text{the}\;\text{three}\;\text{EPICS}\;\text{sessions}\;\times \;100}{\text{Number}\;\text{of}\;\text{eligible}\;\text{participants}\;\text{in}\;\text{the}\;\text{target}\;\text{settings}}$$

In the RE-AIM framework, effectiveness is the impact of the intervention on desired outcomes. For the EPICS cRCT, CRC screening adherence will be based on baseline community CRC screening rates from the Centers for Disease Control and Prevention’s (CDC) Behavioral Risk Factor Surveillance System (BRFSS), post intervention participant self-reported FOBT, flexible sigmoidoscopy and/or colonoscopy, and changes in facilitators’ CRC knowledge, attitudes, and beliefs. Adherence to CRC screening on a total population basis is calculated as:

Number of participants

$$\text{Adherence}\;\text{to}\;\text{CRC}\;\text{screening}=\frac{\text{adherent}\;\text{to}\;\text{CRC}\;\text{screening}\;\text{in}\;\text{the}\;\text{population}}{\text{Estimated}\;\text{target}\;\text{population}\;\text{by}\;\text{setting}}$$

In alignment with Glasgow’s RE-AIM definition, our estimate of effectiveness will be determined using the following formula [24]:

Number of participants in the sample of the target population

$$\text{Estimate}\;\text{of}\;\text{Effectiveness}=\frac{\text{who}\;\text{were}\;\text{Reached}\;\text{and}\;\text{adherent}\;\text{to}\;\text{CRC}\;\text{screening}}{\text{Number}\;\text{of}\;\text{participants}\;\text{in}\;\text{the}\;\text{target}\;\text{population}\;\text{who}\;\text{were}\;\text{reached}}$$

To determine the effectiveness of this dissemination study, we will examine changes in CRC screening adherence rates, based on the pre-intervention and post-intervention in each NBLIC community coalition targeted community. Use of individual outcome measures in evaluating group-level interventions is valid and widely used with an appropriate analysis framework. Our analysis will be based on the principle of intention-to-treat and use the Generalized Estimating Equation (GEE) for modeling the CRC screening adherence in all African Americans, ages 50 to 74 years in the targeted communities pre-intervention and post-intervention. Specifically, the model for the i^th^ community’s j^th^ time point’s k^th^ participant’s adherence status Y_ijk_ will take the form: logit Pr[Y_ijk_ = Adherent] = β_0_ + β_1_ I_{i = community}_ + β_2_ I_{j = Post-intervention}_ + β_3_ I_{i = community_ I_{j = Post-intervention}_ + γ^T^**X** where I_{A}_ is an indicator variable for a set A, **X** is a vector of covariates to be adjusted for, and β’s and γ (a vector communities, in the change in log odds of the CRC screening adherence from pre-intervention to post-intervention. Our statistical inference on β_3_ will be based on the asymptotic robust inference procedure of the GEE method. Special caution will be exercised in the inclusion of covariates **X** into the model and the interpretation of the results, as our study design is a cluster randomized controlled trial.

### Secondary outcome measures

Qualitative and quantitative research methods are useful in gaining insight and understanding of human behavior, and are recommended by Rogers to study the process of an innovation’s adoption. The secondary outcome for the EPICS cRCT is the percentage of clusters and settings implementing the EPICS sessions. In the RE-AIM framework, adoption is concerned with the representativeness of both the setting in which an intervention is conducted and the change agents who deliver the intervention. EPICS implementation is concerned with quality and consistency of the intervention delivery. It refers to the extent to which various components of an intervention are delivered as intended in real-world settings.

The final RE-AIM dimension, maintenance, refers to the extent to which a program or policy becomes institutionalized or part of the routine organizational practices and policies. At the individual level, measuring maintenance requires follow-up contact six-months or more post-intervention. Due to the timing of CRC screening recommendations (*e.g.*, one to ten years), we will not measure individual maintenance, but will focus at the change agent or cluster level, measuring long-term institutional effects. Organizational impact will be measured based on the following calculation, where fidelity is scored 1 to 4 according to the core elements listed in Table 1:

$$\text{Estimate}\;\text{of}\;\text{Impact}=\frac{\%\;\text{clusters}\;\text{adopting}x\%\;\text{implementing}\;\text{x}\%\;\text{institutionalizing}\;\text{EPICS}}{\text{fidelity}\;\text{to}\;\text{core}\;\text{elements}}$$

**Table 1:** EPICS sessions

**#**	**Title**	**Content**
**1**	Introduction to EPICS	This session provides a general overview of colorectal cancer (CRC) facts. Definitions and screening guidelines; fecal occult blood test (FOBT), sigmoidoscopy and colonoscopy; costs; insurance coverage
**2**	Colorectal cancer screening, symptoms and diagnosis	Common symptoms explained. Finding the cause of symptoms through CEA assay, biopsy, x-rays, sigmoidoscopy or colonoscopy. Definition of treatment methods (surgery, chemotherapy, radiation therapy, biological therapy) Clinical Trials; Social support; developing a plan; monitoring success; EPICS colorectal cancer screening goals.
**3**	Maintaining healthy habits	This session encourages participants to incorporate healthier cooking and eating habits into their lifestyles. It also focuses on CRC screening as an important health habit. Incorporating these healthier lifestyles may potentially confound the effect of the intervention on CRC use.

Adoption is the percent of NBLIC community coalitions approached for participation who did, in fact, participate. Adjustments for attrition will be made using intent-to-treat analysis*.* Implementation is the average percent and extent to which core elements are implemented as intended. Institutionalization is the extent to which NBLIC community coalitions continue to offer EPICS post-implementation.

### Cost-effectiveness analysis

The active dissemination strategies used in this project are expected to be more effective in engaging the community to implement EPICS with fidelity and thus more effective in increasing CRC screening rates. However, they will also be more expensive than the passive strategies of dissemination. Therefore, in a cost-effectiveness analysis we will determine whether the increased cost is worth the increased effectiveness. We will compare costs and outcomes of the WA, WA + TA, IP, IP + TA dissemination strategies. The cost-effectiveness analysis will be done from the perspective of a public health agency interested in disseminating EPICS (disseminator). It will thus include costs and benefits of disseminator, community coalitions, and settings. Effectiveness will be measured as described above by the increased adherence to CRC screening. Costs of the dissemination strategies will be collected using the assessment tools described above, plus study records and time logs (for example for costs of web and IP training time of personnel and materials, marketing materials, TA personnel time and expenses). From the NBLIC community coalitions we will collect data on costs incurred to engage community partners. Moreover, via periodic telephone interviews to assess the adherence to EPICS implementation protocol we will also collect information on costs related to recruiting participants and delivering the intervention. Costs of personnel time will be valued using age and gender specific wages as reported by the Bureau of Labor Statistics. Costs and outcomes occurring in year two will be discounted using a 3% discount rate. Costs and outcomes of each dissemination strategy will be compared to first eliminate dominated strategies, *i.e.*, those that are more costly than the next most effective ones. Among those that are not dominated we will calculate Incremental Cost Effectiveness Ratios (ICERs), *i.e.*, the ratio of the costs of each strategy net of those of the next less effective one, by the effectiveness. ICERs will be compared to those of similar interventions to establish cost-effectiveness. In addition, we will obtain measures of efficiency for each strategy, *i.e.*, cost per person screened, cost per community partner engaged, and other similar measures. In sensitivity analyses we will vary inputs of the analysis such as wages of personnel or number of participants per education session, to provide a range of cost-effectiveness values under different assumptions.

### Methods and statistical analysis

Statistical analysis will be completed based on the RE-AIM (Reach, Effectiveness, Adoption, Implementation, Maintenance) framework based on methods as outlined below.

### Reach

Methods determining EPICS’s reach included: establishing inclusion criteria and documenting exclusions, participation rates, dropouts and representativeness; assessing size and characteristics of NBLIC community coalitions and church, clinical and community settings to determine barriers to participation (*e.g.*, infrastructure, time, priorities, etc.); assessing the proportion of eligible participants (African American, age 50 to 74, either gender) per setting consenting participation; documenting the number of community coalitions by characteristics; description of settings (total number of eligible participants); and estimating the number of eligible individuals and completers of EPICS sessions per setting. Proportion of eligible community coalitions and settings aware of EPICS will be calculated and two-group *t* tests and chi-square tests to compare those aware versus not aware will be completed. Analysis of variance will compare the degree of change in EPICS awareness among leaders of community coalitions and settings and the increase in post intervention CRC screening knowledge scores will be determined using a paired *t* test.

### Effectiveness

To determine effectiveness, investigators will: compare CRC screening rates of passive and active dissemination approaches by setting; examine baseline/post CRC screening rates (community coalition catchment area) and compare baseline/post CRC knowledge attitudes; and beliefs of facilitators (training) and participants (EPICS sessions). Effect size will be computed by subtracting mean CRC screening rates in communities aware and not aware of EPICS divided by the pooled standard deviation of CRC screening rates. The proportion of community coalitions and settings aware of EPICS (effectiveness-organizational level) and that deliver it (adoption) will also be determined.

### Adoption

In evaluating adoption, methods include: determining EPICS fit, cost, level of resources and expertise and similarity to current programs; estimating adoption rate (proportion of community coalitions trained that implement EPICS); and documenting reasons for not adopting EPICS (time, total membership or infrastructure, partnerships, program priorities). Descriptive statistics will be used to provide insight into organizational and individual reasons for (not) adopting EPICS. Differences between community coalitions adopting and not adopting EPICS and differences between facilitator characteristics will be analyzed using independent sample *t* tests and chi-square tests.

### Implementation

To evaluate EPICS implementation, the following methods will be employed: train and certify facilitators to deliver educational sessions; implement marketing strategies to enhance visibility; provide on-going technical assistance (TA) to organizations and settings; determine adherence to implementation protocol by passive and active dissemination sites; estimate total number of completers; conduct one-and-one-half day training guided by three modules: principles, procedural and practical knowledge; distribute a monthly newsletter, provide promotional flyers and posters to community coalitions and settings; conduct one-hour monthly teleconferences; assess the nature and quality of TA; measure level of fidelity to delivering sessions as developed; and determine total number of individuals completing the three educational intervention sessions. Descriptive statistics will determine differences in facilitator characteristics, knowledge, attitudes, and beliefs and level of satisfaction with training, the most effective marketing strategies, reasons for not implementing components, duration, and investment and differences facilitator demographic characteristics: education, gender, profession, insurance status, etc. Logistic regression analysis will evaluate variations in the effects of implementation barriers among community coalitions, settings and individual participants.

### Maintenance

To evaluate the final construct in the RE-AIM framework, the following methods will be completed: determine percent of community coalitions indicating that EPICS will become standard practice; measure program feasibility, including cost, time, and human resources for program implementation; determine if EPICS facilitator training and curriculum is offered to additional settings; investigate the extent to which EPICS is integrated into community coalition planned activities; measure enablers and barriers to continued EPICS implementation and plans to continue or ‘intent’ to offer; determine number of clusters and settings who had once offered EPICS but stopped; and determine the extent to which churches, clinical providers and community sites continue EPICS sessions post-implementation. Logistic regression analysis will evaluate variations in the effects of implementation barriers among community coalitions and settings. Descriptive statistics will determine setting by type and number completing facilitator training.

### Trial timeframe

The EPICS cRCT is planned from May 2012 until March 2017. In months one to three, the EPICS Coordinating Center will be established and monthly teleconferences, between the MSM research team and NBLIC community coalitions, will take place. Community coalitions will be randomized into four study arms. Recruitment of community settings (*e.g.*, churches, clinics, and community sites) and potential facilitators by community coalitions and baseline CRC screening rates for each targeted community will also be completed during this time. From months four to seven, facilitator training/certification will be completed, followed by a four-month pilot period (months eight to twelve). The implementation phase, months 13 to 36, includes delivery of three educational sessions and follow-up to determine CRC screening status of 7,200 African Americans in 13 US states to evaluate EPICS adoption and effectiveness. The final 18 months of the active study (months 37–54) will be dedicated to assessing EPICS maintenance. The final six months (54–60) will be reserved for data analysis, publications, and reporting.

### Trial status

The EPICS cRCT completed its pilot phase February 28, 2013. The Implementation Phase will begin May 1, 2013.

## Discussion

Implementing EPICS in partnership with community coalitions, we hypothesized, will result in more rapid adoption than traditional top-down approaches, and that changes in community CRC screening practices are more likely to be sustainable over time. With its national reach, this study has the potential to enhance our understanding of barriers and enablers to the uptake of educational programs aimed at eliminating cancer health disparities.

Glasgow *et al.* at the National Institutes of Health (NIH) have stated: ‘there is a need for research testing approaches to scaling up and sustaining effective interventions’ [25]. This project will help address that need and will focus on the five core values cited by Glasgow *et al.*: rigor and relevance, efficiency, collaboration, improved capacity, and cumulative knowledge. Regarding the first, the project will utilize the rigorous research methods required of NIH-funded studies; but of equal importance, it will test dissemination in settings that are similar to those in communities across the country, where they often serve African Americans. The project is efficient in that it tests several settings simultaneously. It involves widespread collaboration through its reliance on community coalitions that are comprised of healthcare professionals, business people, lay advocates, cancer survivors, and others. The project is building improved capacity in these coalitions; as a consequence, they will be prepared to participate in other cancer prevention studies in the future. The project is the product of cumulative knowledge developed through the original community intervention trial, the local practice demonstration, and the statewide initiative. These multiple iterations made possible the current study. The current study should be the last iteration; at its conclusion, EPICS will be positioned to become a part of standard health promotion practice.

## Abbreviations

ACS: American Cancer Society; BRFSS: Behavioral Risk Factor Surveillance System; CCSIT: Colorectal Cancer Screening Trial; CDC: Centers for Disease Control and Prevention; CRC: Colorectal cancer; cRCT: Cluster randomized controlled trial; EPICS: Educational Program to Increase Colorectal Cancer Screening; DNA: Deoxyribonucleic acid; FOBT: Fecal occult blood test; GCC: Georgia Cancer Coalition; GEE: Generalized Estimating Equation; H1: Hypothesis one; H2: Hypothesis two; H3: Hypothesis three; ICER: Incremental Cost Effectiveness Ratios; IP: In-person; IRB: Institutional Review Board; MOU: Memorandum of Understanding; MSM: Morehouse School of Medicine; NBLIC: National Black Leadership Initiative on Cancer; NCI: National Cancer Institute; RCCG: Regional Cancer Coalitions of Georgia; RE-AIM: Reach, Effectiveness, Adoption, Implementation, Maintenance; RTIPs: Research Tested Intervention Programs; TA: Technical assistance; WA: Web-access.

## Competing interests

Both authors declare that they have no competing interests.

## Authors’ contributions

SAS and DSB, both principal investigators, conceived the study and acquired funding; SAS wrote the first draft of the manuscript. DSB refined the draft and completed the discussion section. Both authors advised on theoretical and methodological issues, providing ongoing critique and have approved the final version of the manuscript.

## References

[B1] American Cancer Society Colorectal cancer 2013 [http://www.cancer.org/cancer/colonandrectumcancer/index]

[B2] Guide to community preventive services[http://www.thecommunityguide.org/]

[B3] CDC behavioral risk factor surveillance system prevalence and trends data [http://apps.nccd.cdc.gov/brfss/page.asp?cat=CCandyr=2009andstate=UB#CC]

[B4] NIH State-of-the-Science Conference Enhancing use and quality of colorectal screening February 2–4, 2010 [http://consensus.nih.gov/2010/images/colorectal/colorectal_panel_stmt.pdf.]23061065

[B5] SEER fact sheets: colon and rectum[http://seer.cancer.gov/statfacts/html/colorect.html.]

[B6] Blumenthal DS Smith SA Majett C Mensah E Impact of three interventions on colorectal cancer screening rates among African Americans Cancer 2010 116 922 929 10.1002/cncr.24842 20052732 PMC2819540

[B7] Israel BA Schulz AJ Parker EA Becker AB **Review of community-based research: assessing partnership approaches to improve** Public Health Ann Rev 1998 19 173 202 10.1146/annurev.publhealth.19.1.173 9611617

[B8] Guide to community preventive services[http://www.thecommunityguide.org/cancer/screening/default.htm]

[B9] Smith SA Blumenthal DS Community health workers support community-based participatory research ethics: lessons learned along the research-to-practice-to-community continuum J Health Care Poor Underserved 2012 23 Suppl 77 88 23124502 10.1353/hpu.2012.0156 PMC3586526

[B10] Smith SA Johnson L Wesley D McCray G Sheats J Blumenthal DS Translation to practice of an intervention to promote colorectal cancer screening among African Americans Clin Transl Sci 2012 5 5 412 415 10.1111/j.1752-8062.2012.00439.x 23067354 PMC3476058

[B11] Sullivan LW Jackson F Sheats JQ Smith SA History of NBLIC Minority Health Today 2000 2 1 10.1023/A:1009522121275

[B12] Moher D Schykz K Altman D The CONSORT statement: revised recommendations for improving the quality of reports of parallel group randomized trials BMC Med Res Methodol 2001 1 2 10.1186/1471-2288-1-2 11336663 PMC32201

[B13] Glasgow RE Marcus AC Bull SS Wilson KM Disseminating effective cancer screening interventions Cancer 2004 101 5 Suppl 1239 1250 15316911 10.1002/cncr.20509

[B14] Merriam SB Caffarella RS Learning in adulthood: a comprehensive guide 1998 San Francisco: Jossey-Bass

[B15] Bero LA Fgilli R Grenshaw JM Closing the gap between research and practice: an overview of systematic reviews of interventions to promote the implementation of research findings BJM 1998 317 465 468 10.1136/bmj.317.7156.465 PMC1113716 9703533

[B16] Rogers EM Diffusion of innovations 1995 New York: Free Press

[B17] Graham ID Tetroe J KT theories research group some theoretical underpinnings of knowledge translation Acad Emerg Med 2007 14 11 936 941 17967955 10.1197/j.aem.2007.07.004

[B18] Glasgow RE Strycker MA King DK Toobert DJ Rahm AK Jex M Nutting PA Robustness of a computer-assisted diabetes self-management intervention across patient characteristics, healthcare settings, and intervention staff Am J Manag Care 2006 12 3 137 145 16524346

[B19] Akers L Gordon JS Andrews JA Barckley M Lichtenstein E Severson HH Cost effectiveness of changing health professionals’ behavior: training dental hygienists in brief interventions for smokeless tobacco cessation Prev Med 2006 43 6 482 487 10.1016/j.ypmed.2006.07.006 16920184

[B20] Kim AE Towers A Renaud J Zhu J Shea JA Galvin R Volpp KG Application of the RE-AIM framework to evaluate the impact of a worksite-based financial incentive intervention for smoking cessation J Occup Environ Med 2012 54 5 610 614 10.1097/JOM.0b013e31824b2171 22476113

[B21] van Acker R de Bourdeaudhuij I de Cocker K Klesges LM Cardon G The impact of disseminating the whole-community project ’10,000 Steps’: a RE-AIM analysis BMC Publ Health 2011 11 3 10.1186/1471-2458-11-3 PMC3022698 21205290

[B22] Ukoumunne OC Gulliford MC Chinn S Sterne JA Burney PG Methods for evaluating area-wide and organization-based interventions in health and health care: a systematic review Health Technol Assess 1999 3 92 10982317

[B23] Bopp M Wilcox S Laken M Hooker SP Saunders R Parra-Medina D Using the RE-AIM framework to evaluate a physical activity intervention in churches Prev Chronic Dis 2007 4 4 PMC2099285 17875262

[B24] Collard DC Chinapaw MJ Verhagen EA van Mechelen W Process evaluation of a school based physical activity related injury prevention programme using the RE-AIM framework BMC Pediatr 2010 10 86 10.1186/1471-2431-10-86 21092316 PMC3004886

[B25] Glasgow RE Klesges LM Dzewaltowski DA Estabrooks PA Vogt TM Evaluating the impact of health promotion programs: using the RE-AIM framework to form summary measures for decision making involving complex issues Health Educ Res 2006 5 688 694 16945984 10.1093/her/cyl081

